# Supplementary material for: The Marginal Ice Zone as a dominant source region of atmospheric mercury during central Arctic summertime
Source: Nat Commun. 2023 Aug 14;14:4887. doi: 10.1038/s41467-023-40660-9 (PMC10425351; doi:10.1038/s41467-023-40660-9)
Supplement: Supplementary file 1 — Supplementary Information [file 41467_2023_40660_MOESM1_ESM.pdf]

Supplementary Information for

The Marginal Ice Zone as a dominant source region of atmospheric mercury during central Arctic summertime

Fange Yue[1], Hélène Angot[2,3,4*], Byron Blomquist[5,6], Julia Schmale[2], Clara J.M. Hoppe[7], Ruibo Lei[8], Matthew D. Shupe[5,6], Liyang Zhan[9], Jian Ren[10], Hailong Liu[11], Ivo Beck[2], Dean Howard[5,6], Tuija Jokinen[12,13], Tiia Laurila[12], Lauriane Quéléver[12], Matthew Boyer[12], Tuukka Petäjä[12], Stephen Archer[14], Ludovic Bariteau[5,6], Detlev Helmig[15], Jacques Hueber[16], Hans-Werner Jacobi[4], Kevin Posman[14], Zhouqing Xie[1,*]

[1]Institute of Polar Environment & Anhui Key Laboratory of Polar Environment and Global Change, Department of Environmental Science and Engineering, University of Science and Technology of China, Hefei, Anhui, 230026, China

[2]Extreme Environments Research Laboratory, École Polytechnique Fédérale de Lausanne (EPFL) Valais Wallis, Sion, Switzerland

[3]Institute for Arctic and Alpine Research (INSTAAR), University of Colorado Boulder, Boulder, CO, United States

[4]Univ. Grenoble Alpes, CNRS, INRAE, IRD, Grenoble INP, IGE, 38000 Grenoble, France

[5]Cooperative Institute for Research in Environmental Sciences, University of Colorado, Boulder, CO, United States

[6]NOAA, Physical Sciences Laboratory, Boulder, CO, United States

[7]Alfred Wegener Institut – Helmholtzzentrum für Polar- und Meeresforschung, Am Handelshafen 12, 27570 Bremerhaven, Germany

[8]Key Laboratory for Polar Science of the MNR, Polar Research Institute of China, Shanghai, China

[9]Third institute of oceanography, Ministry of natural resources, Xiamen, China

[10]Key Laboratory of Marine Ecosystem Dynamics, Second Institute of Oceanography, Ministry of Natural Resources, Hangzhou 310012, China

[11]School of Oceanography, Shanghai Jiao Tong University, Shanghai 200030, China

[12] Institute for Atmospheric and Earth System Research (INAR)/Physics, Faculty of Science, University of Helsinki, Helsinki, Finland

[13]Climate & Atmosphere Research Centre (CARE-C), The Cyprus Institute, Nicosia, Cyprus

[14]Bigelow Laboratory for Ocean Sciences, Boothbay, Maine, United States

[15]Boulder Atmosphere Innovation Research, Boulder, Colorado, United States

[16]JH Atmospheric Instrumentation Design, Boulder CO, United States

**Correspondence to*: Zhouqing Xie (zqxie@ustc.edu.cn) and Hélène Angot (helene.angot@univ-grenoble-alpes.fr)

Supplementary Table 1. Parameters that were tested and used as gaseous elemental mercury (GEM) covariates in the generalized additive model (GAM) in this study.

Variable name	Data state	F values	R^2	AIC values	Description
Open-water	Used	117.589	0.461	-907.51	Open-water fraction at the Polarstern position
CO	Used	5.998	0.491	-1030.3	Carbon monoxide
WS	Used	26.11	0.523	-1169	Wind speed
Temp	Used	46.215	0.575	-1423.1	Air temperature
Traj48h	Used	24.295	0.614	-1627.7	The distance of the endpoint of each 48 h trajectory from the corresponding cruise observation location
P	Used	23.247	0.632	-1731.8	Atmospheric pressure
RD	Tested	3.682	0.636	-1749.3	Solar radiation
PBLH	Tested	7.613	0.649	-1820.9	Planetary boundary layer height
LH	Tested	7.753	0.66	-1886.2	Latent heat flux
RH	Tested	7.475	0.668	-1930.9	Relative humidity

Supplementary Fig.1. Time series for measured gaseous elemental mercury (GEM), sulfur dioxide (SO_2) and carbon monoxide (CO) concentrations, and solar radiation (RD) during the period of GEM summertime maximum. The yellow shaded area shows the observed highest hourly GEM concentration during the whole observation period.

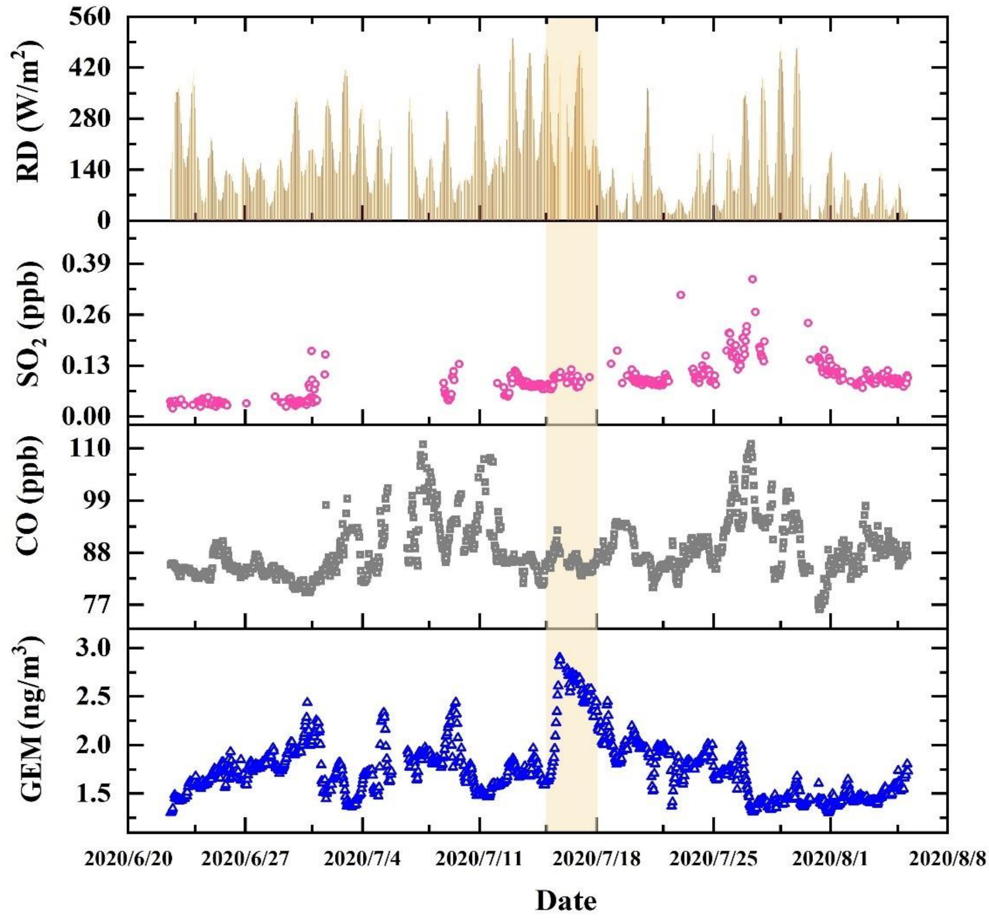

Supplementary Fig.2. The 7-day backward trajectory inversion results during the whole observation period. The trajectory results were created using the HYSPLIT transport and dispersion model from NOAA Air Resources Laboratory (ARL). The red dots in the map represent cities. The figure was generated using Meteoinfo software(http://www.meteothink.org/)[5].

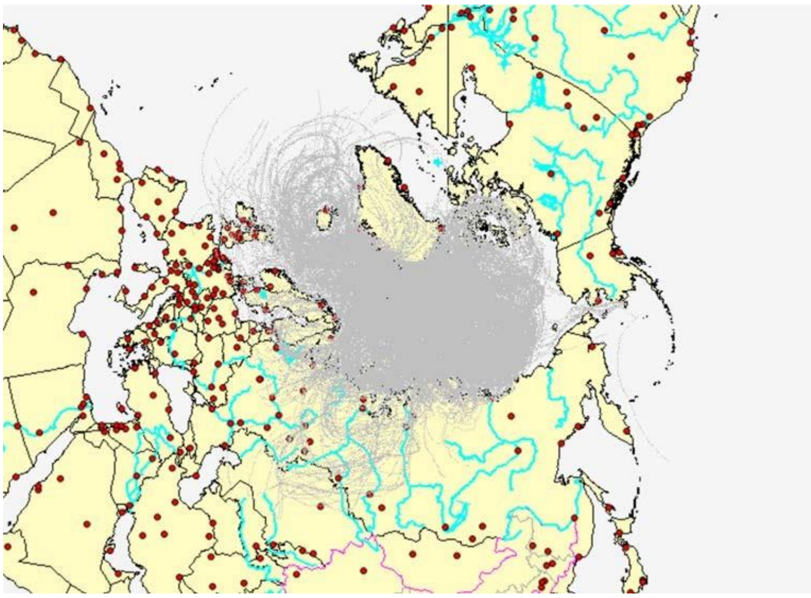

Supplementary Fig.3. Spatial distribution of remote sensing sea-ice concentration during the summertime gaseous elemental mercury (GEM) peaks. Spatial distribution of AMSR2 (Advanced Microwave Scanning Radiometer) remote sensing sea-ice concentration by satellite remote sensing during the summertime GEM peaks (on July 16, 2020). The black box marks the site where the GEM peaks were observed. The figure was directly generated online (https://seaice.uni-bremen.de/sea-ice-concentration/amsre-amsr2)[6].

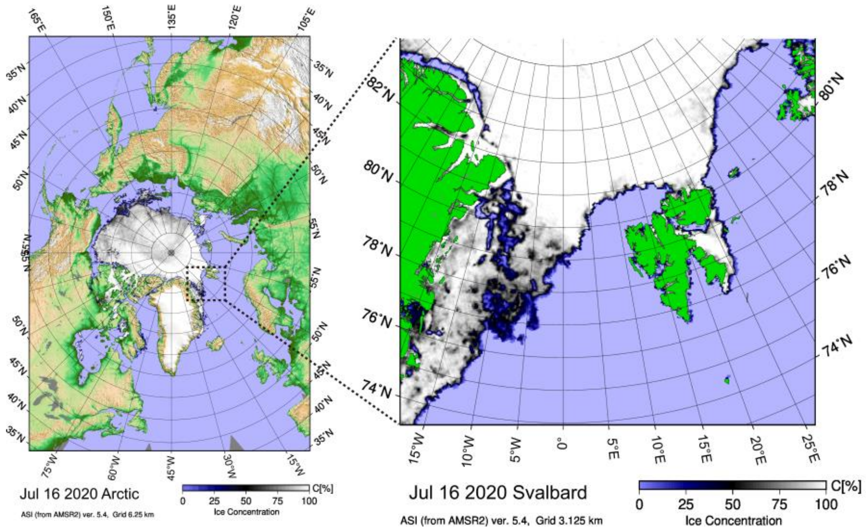

Supplementary Fig.4. Spatial distribution of remote sensing particulate organic carbon (POC) concentration in surface seawater during the summertime gaseous elemental mercury (GEM) peaks. Spatial distribution of Aqua-MODIS (Moderate Resolution Imaging Spectroradiometer) remote sensing 8 days-averaged particulate organic carbon (POC) concentration in surface seawater by satellite remote sensing during the summertime GEM peaks (from July 11 to 18, 2020). The figure was freely generated using Panoply software (https://www.giss.nasa.gov/tools/panoply/).

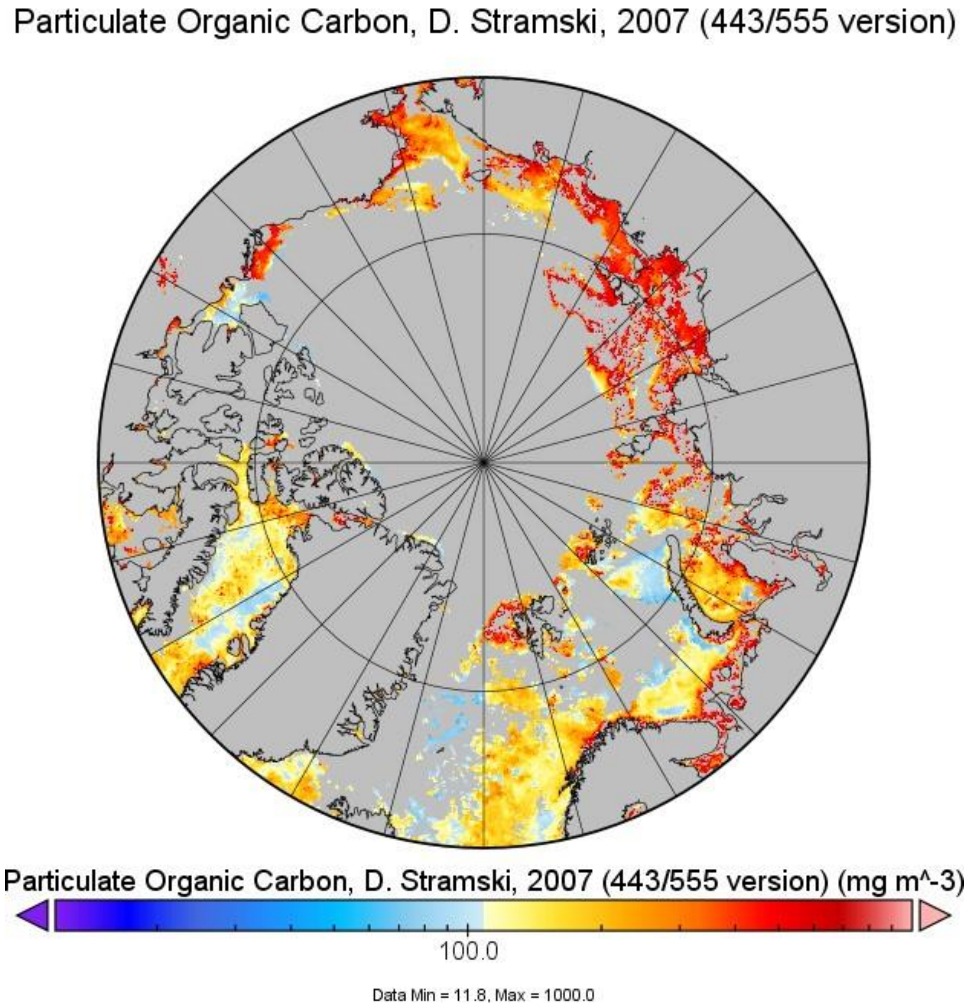

Supplementary Fig.5. The latitudinal and the corresponding time series of gaseous elemental mercury (GEM) concentrations during the ship transited northward from the marginal ice zone (MIZ) to the North Pole. The color bar indicated the spatial distribution of GEM concentrations, and the arrow marked the travelling direction of the vessel. The bottom panel of figure was generated using Ocean Data View (https://odv.awi.de/)[7].

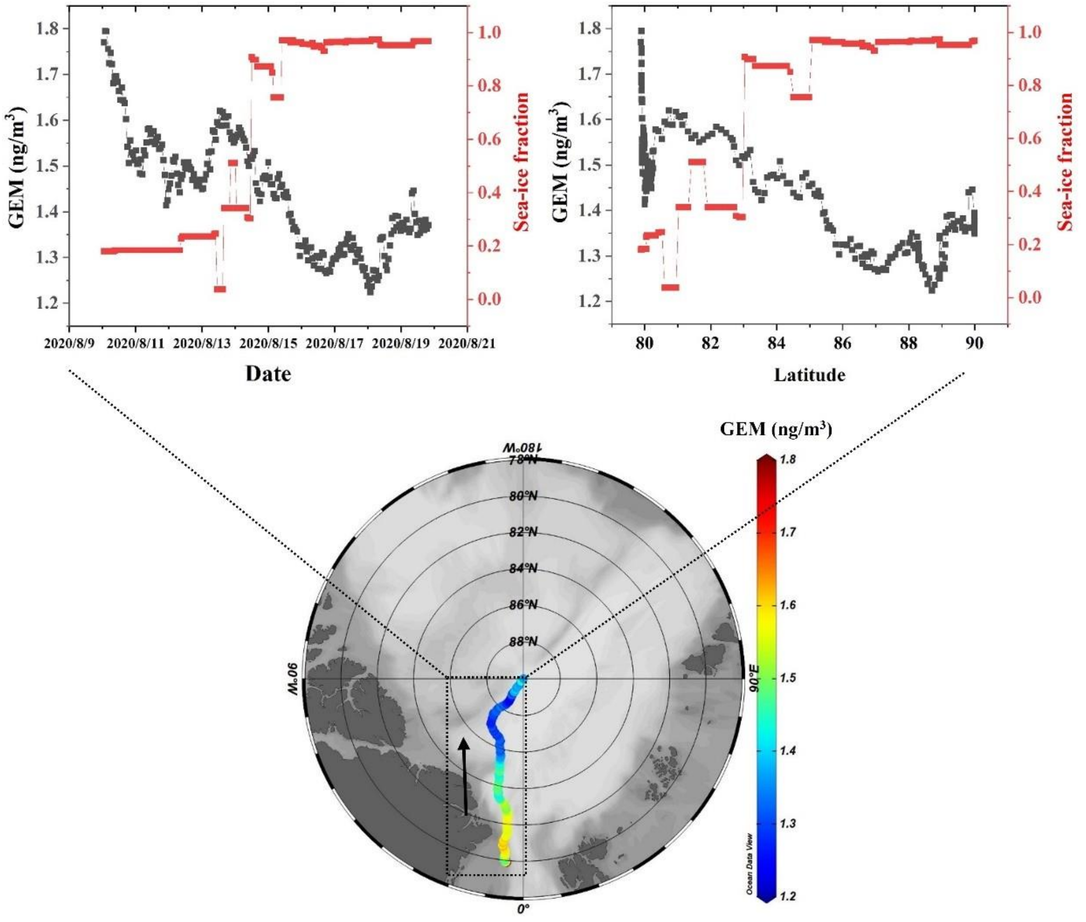

Supplementary Fig.6. Spline of gaseous elemental mercury (GEM) to various meteorological parameters. Spline of gaseous elemental mercury (GEM) to (a) wind speed (WS), (b) temperature (Temp), and (c) surface air pressure (P).

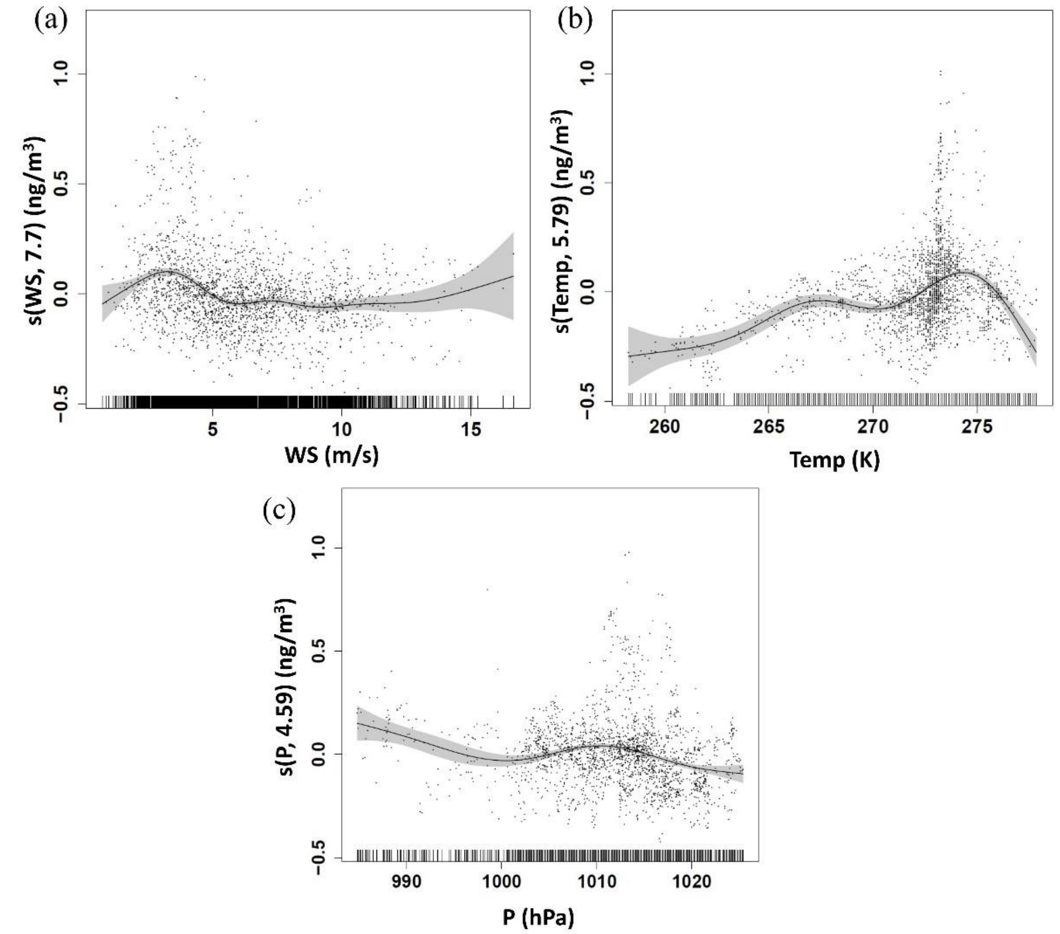

Supplementary Fig.7. Potential source contribution function (PSCF) analysis of air temperature (Temp) with different ranges. Potential source contribution function (PSCF) analysis of air temperature (Temp) with (a) Temp in the range of 260-275k, and (b) with Temp larger than 275k. The red boxes in (a) and (b) indicate the Marginal Ice Zone (MIZ). The figure was generated using Meteoinfo software (http://www.meteothink.org/)[5].

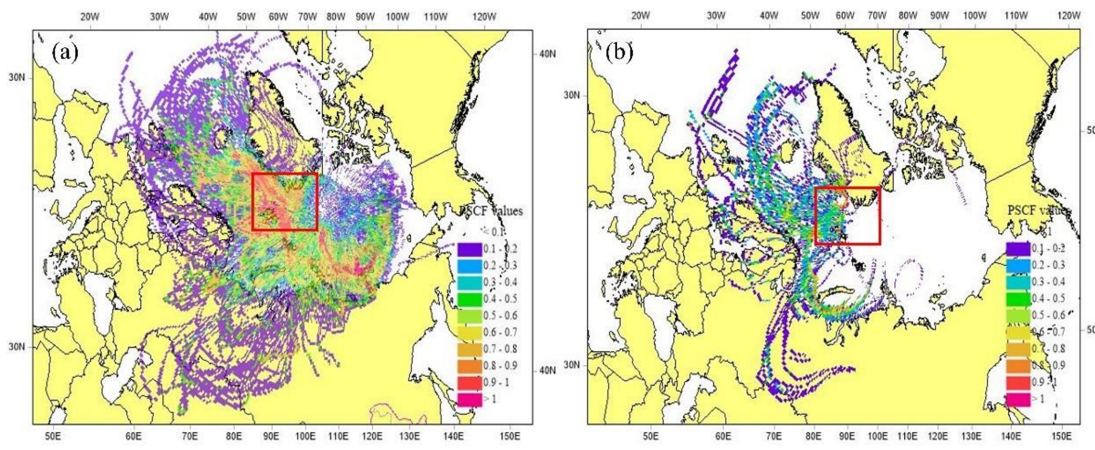

Supplementary Fig.8. The weather map of 50m wind speed (50m Winds) and sea level pressure (SLP) during the gaseous elemental mercury (GEM) peaks. The weather map of 50m wind speed (50m Winds) and sea level pressure (SLP) during the GEM peaks (2020/7/16 6:00). The map was generated online (https://fluid.nccs.nasa.gov/reanalysis/classic_merra2)[8]. The red box marked the region where the highest hourly GEM concentration (which was marked in yellow in supplementary Fig.3 and can be seen in Fig.1a) was occurred.

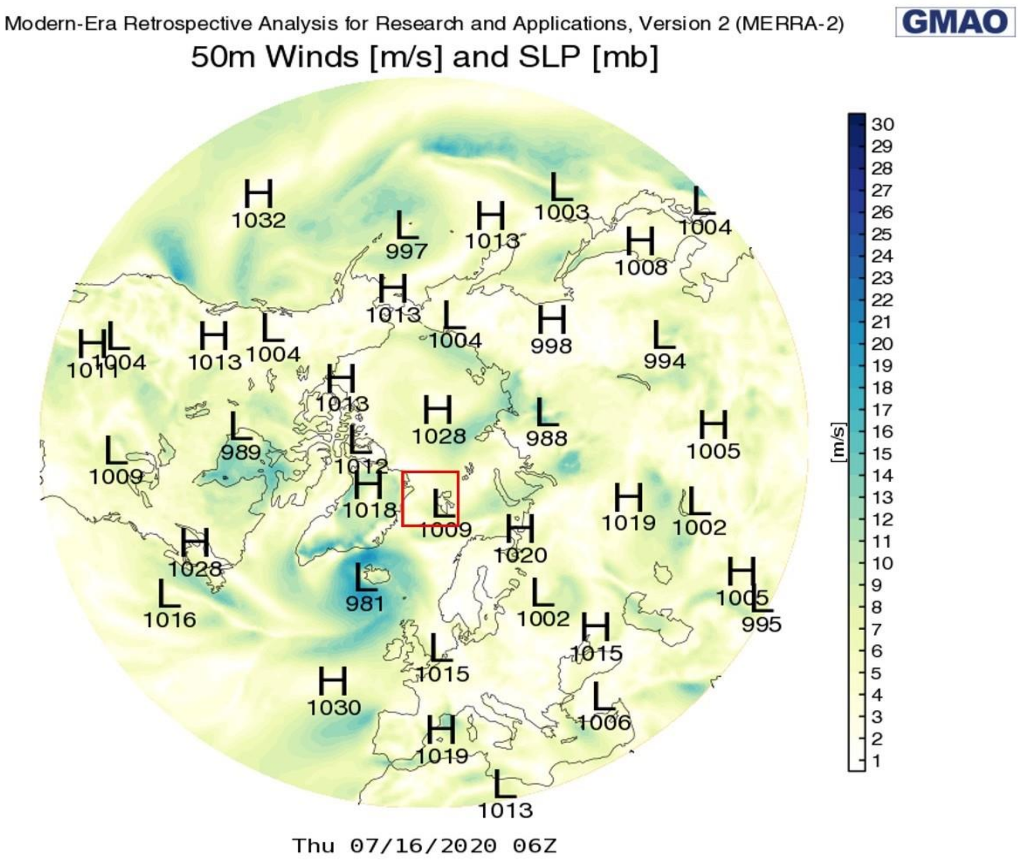

Supplementary Fig.9. Spatial distribution and the location information of observed gaseous elemental mercury (GEM) during the summer legs of the Multidisciplinary drifting Observatory for the Study of Arctic Climate (MOSAiC) expedition. Spatial distribution of observed gaseous elemental mercury (GEM) in the Arctic Ocean during the summer legs (June–September) of the MOSAiC expedition. The grey boxes mark the corresponding locations and date during the expedition. The figure was generated using Ocean Data View (https://odv.awi.de/)[7].

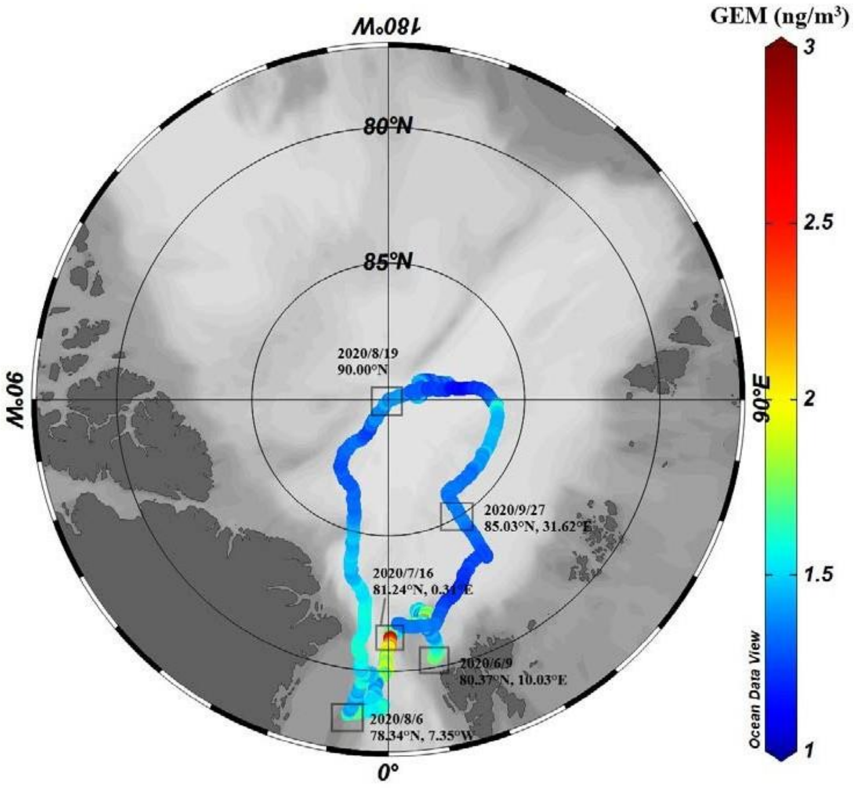

Supplementary Fig.10. Model validation of generalized additive model (GAM) method. (a) The Q-Q plot of residuals against normal distribution, (b) the histogram of residuals, (c) the scatterplot of residuals against linear predictor, and (d) the relationship between the Akaike Information Criterion (AIC values) values and the corresponding GAM fitting R^2 values.

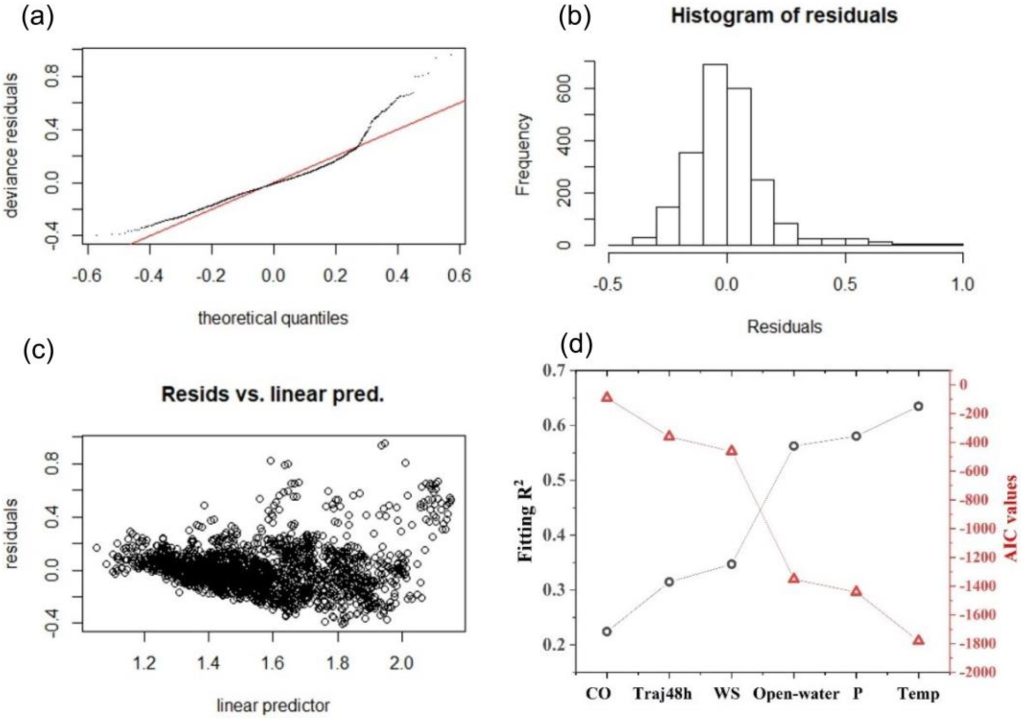

Supplementary Fig.11. Results of generalized additive model (GAM) simulation. (a) GAM fitted gaseous elemental mercury (GEM) (GAM, y-axis) vs. observed GEM (Obs, x-axis), (b) GAM fitted GEM (GAM, x-axis) vs. the 5-fold cross-validation GEM results (5-cv, y-axis), and (c) time series of observed GEM (black dotted line) and simulated GEM through GAM (red dotted line) during the whole observation period (June 9 – September 30, 2020).

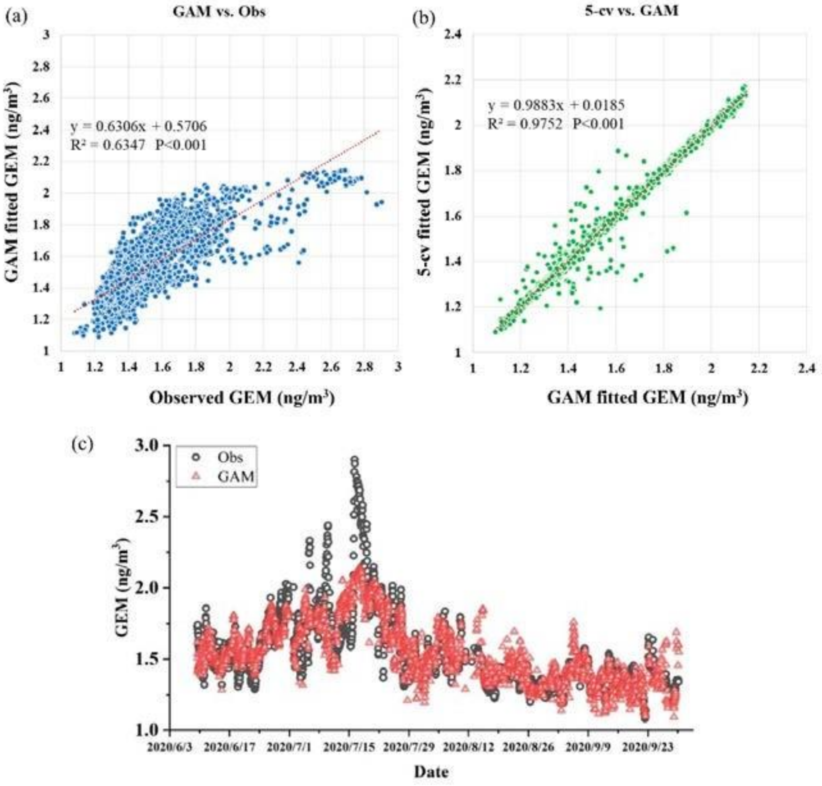

Supplementary Fig.12. Distribution histogram of all the 168h-trajectories' heights used in the potential source contribution function (PSCF) analysis. Distribution histogram of all the 168h-trajectories' transmission heights used in the PSCF analysis in this study. The bar chart shows the data amount of each range of trajectory's height, and the blue line chart shows the proportion of the corresponding data amount of each range of trajectory's height in the total data amount.

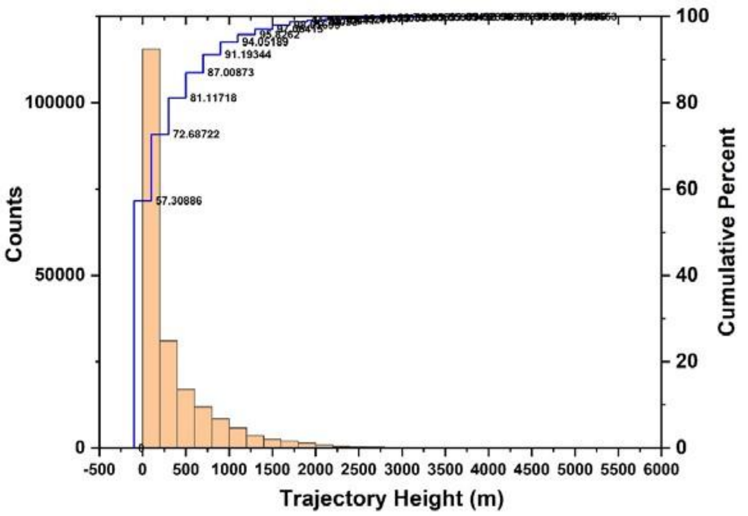

Supplementary Discussion

Estimation of the flux rate of GEM from the MIZ

To estimate the flux rate of GEM from the MIZ between Greenland and Svalbard that is necessary to explain the increase in GEM concentration within the Arctic Ocean boundary layer from 1.3 ng/m^3 to 1.8 ng/m^3, the following assumptions are made here:

(1) Neglect other sources of GEM in the Arctic Ocean and the GEM exchange between the Arctic Ocean and other areas;

(2) The GEM emitted from the MIZ will affect its nearby Arctic Ocean area that is north of 70 °N, as the summertime GEM maximum phenomenon was mainly observed at high-latitude (north of 70 °N) Arctic stations (e.g. Zeppelin, Alert, Villum stations), while low-latitude Arctic stations (e.g. Andøya) observed no apparent GEM maximum phenomenon [1]. In this study, the speculated range of the Arctic Ocean area that the GEM evasion from the MIZ between Greenland and Svalbard would influence is shown in Supplementary Fig.13 in the supporting information (the fan-shaped area with red border is the range of influence, and the triangle area with yellow border is the MIZ that serves as the GEM source):

Supplementary Fig.13. The speculated range of Arctic Ocean area that gaseous elemental mercury (GEM) evasion from the marginal ice zone (MIZ) between Greenland and Svalbard would influence. The fan-shaped area with red border is the range of influence, and the triangle area with yellow border is the MIZ that is served as GEM source. The figure was generated using Drawing 3D software in windows computer system. The corresponding map was directly generated online (https://seaice.uni-bremen.de/sea-ice-concentration/amsre-amsr2)[6].

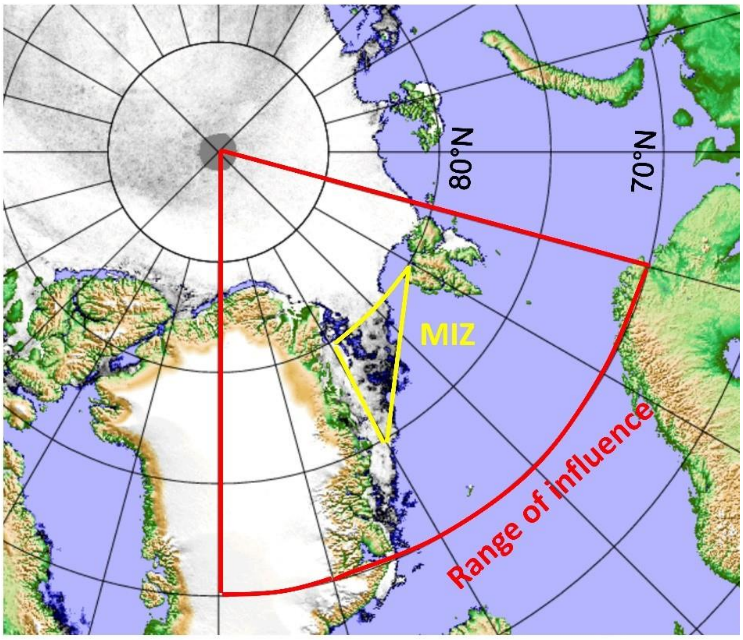

By geometric calculation, the area of MIZ that emits GEM accounts for about 6.25% of its range of influence in the Arctic Ocean.

(3) Based on the time series of GEM in this study, in which the GEM concentration showed apparent increases during three periods in July (from about 2020/6/19 to 2020/7/2, from 2020/7/4 to 2020/7/9, and from 2020/7/11 to 2020/7/18, see Supplementary Fig.14) and mainly occurred in the MIZ, we set the duration of GEM evasion in the MIZ as 20 days.

Supplementary Fig.14. The time series of marginal ice zone (MIZ) during the summer legs (June–September) of the Multidisciplinary drifting Observatory for the Study of Arctic Climate (MOSAiC) expedition. The period of summertime gaseous elemental mercury (GEM) peaks in July is marked with red box and the corresponding daily time series.

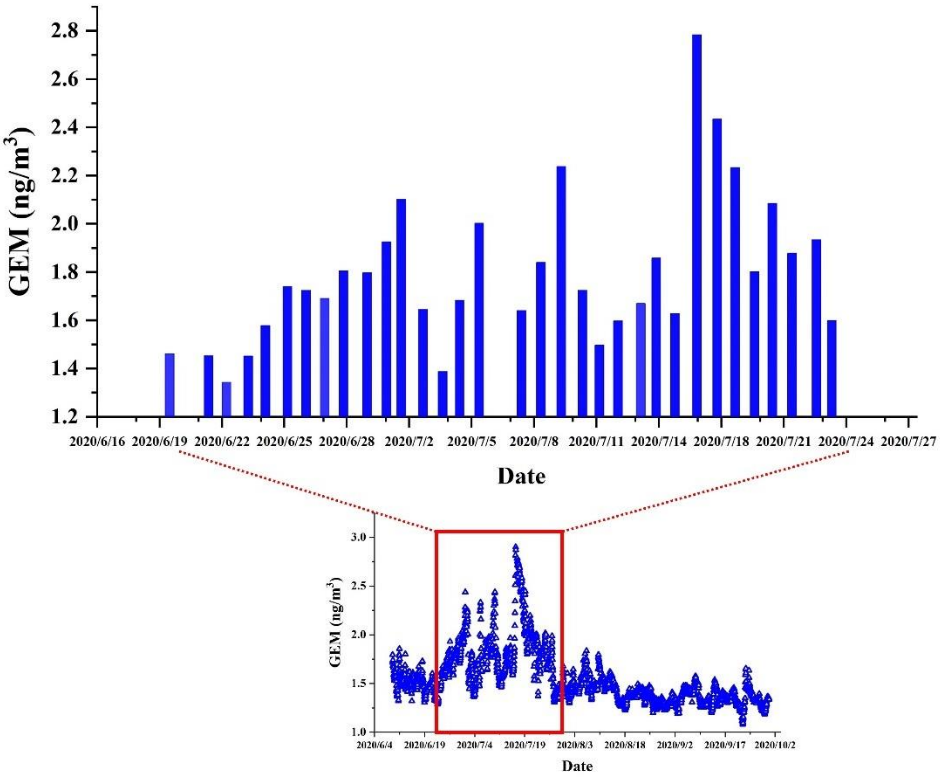

Based on the assumptions above, the GEM flux rate can be calculated using the following equation, according to the principle that mercury evaded from the MIZ in summer leads to the increase in observed GEM concentration in its range of influence in the Arctic Ocean:

$$\text{Flux} \times 0.0625S \times 20 = (1.80 - 1.30) \times S \times \text{PBLH}$$

Where Flux is GEM evasion flux rate (ng m^{-2} day^{-1}) from the MIZ between Greenland and Svalbard; S is the range of influence (km^2) in the Arctic Ocean by GEM evasion from the MIZ; 1.80 (ng/m^3) and 1.30 (ng/m^3) are July peak concentration and September background concentration of GEM in the Arctic respectively; 0.0625 is the area fraction of the MIZ to its range of influence in the Arctic Ocean; and PBLH is the planetary boundary layer height (m) of the Arctic Ocean. Here we set this value as the July average (140m) during the MOSAiC cruise. The calculated flux is 56 ng m^{-2} day^{-1}, which is more than twice the flux in the Arctic ice-free open ocean (< 24 ng m^{-2} day^{-1}) [2, 3], while lower than that observed in coastal areas in the Canadian Arctic Archipelago (~130 ng m^{-2} day^{-1})[4]. This result is just a preliminary estimation, and further corresponding field observation studies and model simulation studies are needed in the future.

Supplementary References

1. Angot H, *et al.* Chemical cycling and deposition of atmospheric mercury in polar regions: review of recent measurements and comparison with models. *Atmos Chem Phys* **16**, 10735-10763 (2016).
2. DiMento BP, Mason RP, Brooks S, Moore C. The impact of sea ice on the air-sea exchange of mercury in the Arctic Ocean. *Deep Sea Research Part I: Oceanographic Research Papers* **144**, 28-38 (2019).
3. Andersson ME, Sommar J, Gårdfeldt K, Lindqvist O. Enhanced concentrations of dissolved gaseous

mercury in the surface waters of the Arctic Ocean. *Marine Chemistry* **110**, 190-194(2008).

4. Kirk JL, St. Louis VL, Hintelmann H, Lehnherr I, Else B, Poissant L. Methylated Mercury Species in Marine Waters of the Canadian High and Sub Arctic. *Environmental Science & Technology* **42**,8367-8373 (2008).
5. Wang, YQ. An Open Source Software Suite for Multi-Dimensional Meteorological Data Computation and Visualisation. *Journal of Open Research Software*, 7(1), p.21. doi: http://doi.org/10.5334/jors.267 (2019)
6. Spreen G, Kaleschke L, Heygster G. Sea ice remote sensing using AMSR-E 89 GHz channels. *J. Geophys. Res.* **113**, C02S03, doi:10.1029/2005JC003384 (2008).
7. Schlitzer, R. *Ocean Data View*. https://odv.awi.de/ (2021).
8. Gelaro R, *et al.* The Modern-Era Retrospective Analysis for Research and Applications, Version 2 (MERRA-2). *Journal of Climate* **30**, 5419-5454 (2017).
